# *Vital Signs*: Burden and Prevention of Influenza and Pertussis Among Pregnant Women and Infants — United States

**DOI:** 10.15585/mmwr.mm6840e1

**Published:** 2019-10-11

**Authors:** Megan C. Lindley, Katherine E. Kahn, Barbara H. Bardenheier, Denise V. D’Angelo, Fatimah S. Dawood, Rebecca V. Fink, Fiona Havers, Tami H. Skoff

**Affiliations:** ^1^Immunization Services Division, National Center for Immunization and Respiratory Diseases, CDC; ^2^Leidos, Atlanta, Georgia; ^3^Division of Reproductive Health, National Center for Chronic Disease Prevention and Health Promotion, CDC; ^4^Influenza Division, National Center for Immunization and Respiratory Diseases, CDC; ^5^Abt Associates, Inc., Cambridge, Massachusetts; ^6^Division of Bacterial Diseases, National Center for Immunization and Respiratory Diseases, CDC.

## Abstract

**Introduction:**

Vaccinating pregnant women with influenza vaccine and tetanus toxoid, reduced diphtheria toxoid, and acellular pertussis vaccine (Tdap) can reduce influenza and pertussis risk for themselves and their infants.

**Methods:**

Surveillance data were analyzed to ascertain influenza-associated hospitalization among pregnant women and infant hospitalization and death associated with influenza and pertussis. An Internet panel survey was conducted during March 27–April 8, 2019, among women aged 18–49 years who reported being pregnant any time since August 1, 2018. Influenza vaccination before or during pregnancy was assessed among respondents with known influenza vaccination status who were pregnant any time during October 2018–January 2019 (2,097). Tdap receipt during pregnancy was assessed among respondents with known Tdap status who reported a live birth by their survey date (817).

**Results:**

From 2010–11 to 2017–18, pregnant women accounted for 24%–34% of influenza-associated hospitalizations per season among females aged 15–44 years. From 2010 to 2017, a total of 3,928 pertussis-related hospitalizations were reported among infants aged <2 months (annual range = 262–743). Maternal influenza and Tdap vaccination coverage rates reported as of April 2019 were 53.7% and 54.9%, respectively. Among women whose health care providers offered vaccination or provided referrals, 65.7% received influenza vaccine and 70.5% received Tdap. The most commonly reported reasons for nonvaccination were believing the vaccine is not effective (influenza; 17.6%) and not knowing that vaccination is needed during each pregnancy (Tdap; 37.9%), followed by safety concerns for the infant (influenza =15.9%; Tdap = 17.1%).

**Conclusions and Implications for Public Health Practice:**

Many pregnant women do not receive the vaccines recommended to protect themselves and their infants, even when vaccination is offered. CDC and provider organizations’ resources are available to help providers convey strong, specific recommendations for influenza and Tdap vaccination that are responsive to pregnant women’s concerns.

## Introduction

Pregnancy confers an increased risk for hospitalization with influenza; one analysis estimated a 2.4 greater odds of influenza-associated hospitalization among pregnant women compared with nonpregnant patients (*1*). Influenza is also dangerous for infants aged <6 months, who have the highest incidence of influenza-associated hospitalizations and highest influenza-associated mortality risk among children (*2*). Similarly, pertussis morbidity and mortality are highest among infants aged <1 year, who have the highest per-population disease and hospitalization incidence and account for 88% of reported pertussis deaths (*3*). Infants routinely receive their first doses of pertussis-containing vaccine at age 2 months and influenza vaccine at age 6 months (*4*).

Vaccinating pregnant women with influenza vaccine and Tdap can provide their infants with transplacentally transferred passive immunity against influenza and pertussis during the first few months of life and also reduce women’s own risk for infection (*5*–*7*). The Advisory Committee on Immunization Practices (ACIP) recommends that all women who are or will be pregnant during influenza season receive influenza vaccination, which can be administered anytime during pregnancy (*8*). ACIP also recommends that women receive a dose of Tdap during each pregnancy, preferably during the early part of gestational weeks 27–36 (*9*). CDC analyzed influenza and pertussis data from national surveillance systems to assess disease burden* among pregnant women and infants and estimated maternal influenza and Tdap vaccination coverage using panel survey data.

## Methods

Data from the Influenza Hospitalization Surveillance Network (FluSurv-NET) and the Influenza-Associated Pediatric Mortality Surveillance System^†^ for the 2010–11 through 2017–18 influenza seasons were analyzed to quantify the proportion of influenza-associated hospitalizations among females aged 15–44 years that occurred among pregnant women and the number of influenza-associated hospitalizations per 100,000 and influenza-associated mortality among infants aged <6 months. Data from the National Notifiable Diseases Surveillance System (NNDSS)^§^ for 2010–17 were analyzed to obtain pertussis case counts, hospitalization proportion (calculated among the 64% of infants with known outcome), and mortality in infants aged <2 months.

An Internet panel^¶^ survey was conducted to estimate influenza and Tdap vaccination coverage among pregnant women (*10*); female panel members aged 18–49 years living in the United States were invited via e-mail or through a link on the panel website to access the survey site and complete screening questions. The survey was fielded during March 27–April 8, 2019, among women aged 18–49 years who reported being pregnant any time since August 1, 2018. Among 20,315 women who entered the survey site, 2,762 reported being eligible; 2,626 completed the survey (cooperation rate = 95.1%).** Data were weighted to reflect age, race/ethnicity, and geographic distribution of the U.S. population of pregnant women (*10*).

Influenza vaccination coverage was calculated among 2,097^††^ women who reported being pregnant any time during October 2018–January 2019; those reporting vaccination before or during pregnancy since July 1, 2018, were considered vaccinated. Report of receipt of Tdap at any point during pregnancy was assessed among 817 women who knew their Tdap vaccination status during their recent pregnancy and reported a live birth by their survey date^§§^; women excluded from Tdap coverage analyses differed on several factors from those included. Pregnancy and vaccination status were self-reported and not verified via medical record review. Receipt of both recommended vaccines was calculated among the Tdap analytic sample (817). Receipt of each vaccine was examined by maternal age, race/ethnicity, education, marital status, employment status, poverty status, insurance type, and residency by U.S. Census region and rurality. Influenza vaccination coverage was calculated by reported number of provider visits since July 2018 and presence of medical condition(s) other than pregnancy indicating increased risk for influenza complications; Tdap vaccination coverage by provider visits was not calculated as reported visits could not be attributed to the specific window (27–36 weeks gestation) during which Tdap is recommended. Receipt of a vaccination offer or referral from a health care provider was calculated and vaccination coverage among women who received an offer or referral was estimated for all demographic subgroups. Because the survey was conducted among a nonprobability sample, statistical significance cannot be inferred. Differences of ≥5 percentage points between proportions compared are noted.^¶¶^

## Results

**FIGURE 1:**
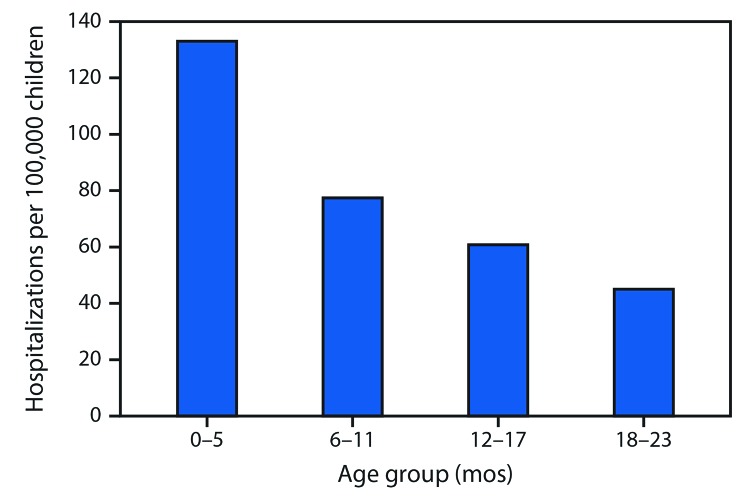
Average number of influenza-associated hospitalizations per 100,000 children aged 0–23 months — Influenza Hospitalization Surveillance Network (FluSurv-NET), United States, 2010–11 through 2017–18 influenza seasons

**FIGURE 2:**
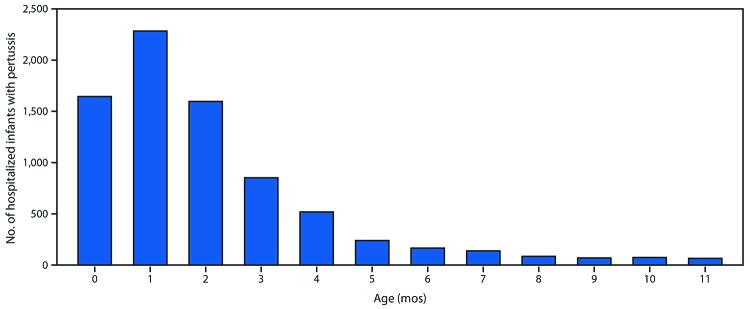
Number of infants with pertussis who were hospitalized, by age in months (N = 7,731) — National Notifiable Diseases Surveillance System, United States, 2010–2017

During the 2010–11 through 2017–18 influenza seasons, 2,341 influenza-associated hospitalizations among pregnant women were reported to FluSurv–NET (seasonal range = 84–523). Pregnant women accounted for 24%–34% of reported influenza-associated hospitalizations per season among females aged 15–44 years with known pregnancy status.*** During the same period, the average influenza-associated hospitalization rate per season among infants aged <6 months was 133.0 per 100,000 with lower rates in older age groups (Figure 1); 100 laboratory-confirmed influenza-associated deaths among infants aged <6 months were reported (seasonal range = 6–19). From 2010 to 2017, pertussis was reported in 27,370 infants aged <12 months; 9,199 cases (33.6%) occurred among infants aged <2 months. Among 7,731 infant pertussis hospitalizations during 2010–17, a total of 3,928 (50.8%) were among infants aged <2 months (Figure 2). During the same period, infants aged <2 months accounted for 69% (77) of NNDSS-reported pertussis deaths.

In the Internet panel survey, 53.7% of eligible respondents reported influenza vaccination before or during pregnancy, and 54.9% reported Tdap vaccination during pregnancy (Table 1). Receipt of both influenza vaccine and Tdap was reported by 34.8% of 817 women with a recent live birth. For both vaccines, vaccination coverage was lower among non-Hispanic black (black) women and women who had less than a college education, were unmarried, lived below the poverty line, lived in the South, were publicly insured, and did not report a vaccination offer or referral from a health care provider than was coverage among referent groups. Influenza vaccination coverage was lower among nonworking women; Tdap coverage was lower among working women. Influenza vaccination coverage was also lower among uninsured women and those with five or fewer provider visits since July 2018. For Tdap, but not for influenza vaccination, Hispanic women had lower coverage, and women aged 18–34 years had higher coverage than did referent groups.

**TABLE 1:** Influenza vaccination and tetanus toxoid, reduced diphtheria toxoid, and acellular pertussis vaccine (Tdap) coverage among pregnant women, by selected characteristics — Internet panel survey, United States, March–April 2019

Characteristic	Influenza		Tdap	
	No. (weighted %)	% (weighted) vaccinated	No. (weighted %)	% (weighted) vaccinated
**Total**	**2,097 (100)**	**53.7**	**817 (100)**	**54.9**
**Age group (yrs)**				
18–24	450 (25.8)	52.9	155 (23.6)	57.9*
25–34	1,165 (54.3)	53.2	480 (57.8)	57.5*
35–49^†^	482 (19.9)	56.2	182 (18.6)	43.1
**Race/Ethnicity^§^**				
White, non-Hispanic^†^	1,262 (49.7)	57.0	542 (55.8)	61.4
Black, non-Hispanic	239 (19.5)	38.0*	87 (18.8)	37.7*
Hispanic	372 (23.1)	57.3	113 (18.6)	51.4*
Other, non-Hispanic	224 (7.7)	61.7	75 (6.9)	58.5
**Education**				
Less than high school diploma	526 (27.0)	46.1*	205 (25.6)	49.3*
Some college, no degree	484 (23.5)	47.9*	206 (26.3)	55.6*
College degree (2- or 4-year)	838 (38.4)	60.0	314 (37.9)	56.7
More than college degree^†^	249 (11.1)	63.0	92 (10.2)	60.6
**Marital status^¶^**				
Married^†^	1,231 (54.9)	62.4	547 (62.0)	58.3
Unmarried	865 (45.1)	43.1*	270 (38.0)	49.4*
Employment status**				
Working^†^	1,178 (56.2)	57.8	396 (48.5)	52.1
Not working	919 (43.8)	48.5*	421 (51.5)	57.5*
**Poverty status^††^**				
At or above poverty^†^	1,609 (75.3)	57.5	624 (75.6)	56.9
Below poverty	485 (24.7)	42.4*	192 (24.4)	49.5*
**Area of residence^§§^**				
Nonrural^†^	1,691 (82.8)	54.6	638 (79.9)	55.2
Rural	406 (17.2)	49.7	179 (20.1)	53.8
**Region** ^¶¶^				
Northeast^†^	342 (17.8)	56.4	126 (17.2)	56.5
Midwest	488 (20.2)	56.0	206 (21.7)	59.4
South	861 (38.0)	50.0*	335 (37.8)	51.4*
West	406 (23.9)	55.9	150 (23.3)	55.3
Prenatal insurance status***				
Private/Military^†^	1,042 (47.3)	62.0	410 (47.7)	61.2
Public	968 (48.7)	47.5*	389 (49.8)	50.4*
Uninsured	87 (4.0)	31.0*	<30 (—^†††^)	—^†††^
**Provider vaccination recommendation/offer^§§§^**				
Offered or referred^†^	1,523 (73.3)	65.7	624 (76.0)	70.5
Recommended, no offer or referral	153 (7.1)	35.9*	43 (5.8)	19.5*
No recommendation	391 (19.6)	18.5*	150 (18.1)	1.0*
**No. of provider visits since July 2018**				
None	30 (1.7)	20.3*	N/A	N/A
1–5	395 (18.5)	46.3*	N/A	N/A
6–10	784 (37.1)	55.4	N/A	N/A
>10^†^	888 (42.7)	56.8	N/A	N/A
**High-risk condition for influenza^¶¶¶^**				
Yes^†^	895 (48.3)	56.3	N/A	N/A
No	979 (51.7)	52.5	N/A	N/A

**Abbreviation:** N/A = not applicable.

* ≥5 percentage-point difference compared with referent group.

^†^ Referent group for comparison within subgroups.

^§^ Race/ethnicity was self-reported. Women identified as Hispanic might be of any race. The “other” race category included Asians, American Indians or Alaska Natives, Native Hawaiians or other Pacific Islanders, and women who selected “other” or multiple races.

^¶^ Excludes one woman who did not report marital status.

** Women who were employed for wages and self-employed were categorized as working; those who were out of work, homemakers, students, retired, or unable to work were categorized as not working.

^††^ Poverty status was defined based on the reported number of people and children living in the household and annual household income, according to U.S. Census poverty thresholds (https://www.census.gov/data/tables/time-series/demo/income-poverty/historical-poverty-thresholds.html).

^§§^ Rurality was defined using ZIP codes where >50% of the population resides in either a nonmetropolitan county and/or a rural U.S. Census tract, according to the Health Resources and Services Administration’s definition of rural population (https://www.hrsa.gov/rural-health/about-us/definition/index.html).

^¶¶^ *Northeast*: Connecticut, Maine, Massachusetts, New Hampshire, New Jersey, New York, Pennsylvania, Rhode Island, and Vermont. *Midwest*: Illinois, Indiana, Iowa, Kansas, Michigan, Minnesota, Missouri, Nebraska, North Dakota, Ohio, South Dakota, and Wisconsin. *South*: Alabama, Arkansas, Delaware, District of Columbia, Florida, Georgia, Kentucky, Louisiana, Maryland, Mississippi, North Carolina, Oklahoma, South Carolina, Tennessee, Texas, Virginia, and West Virginia. *West*: Alaska, Arizona, California, Colorado, Hawaii, Idaho, Montana, Nevada, New Mexico, Oregon, Utah, Washington, and Wyoming.

*** Women pregnant on their survey date were asked about current insurance; women who had already delivered were asked about insurance “during your most recent pregnancy.” Women considered to have public insurance selected at least one of the following when asked what kind of medical insurance they had: Medicaid, Medicare, Indian Health Service, state-sponsored medical plan, or other government plan. Women considered to have private/military insurance selected private medical insurance and/or military medical insurance and did not select any type of public insurance.

^†††^ Estimates with sample size <30 are not reported.

^§§§^ Referral is defined as a “yes” response to the question “Did any doctor, nurse, or medical professional suggest that you go someplace else to get the [flu/Tdap] vaccination?”

^¶¶¶^ Conditions other than pregnancy associated with increased risk for serious medical complications of influenza include chronic asthma, a lung condition other than asthma, a heart condition, diabetes, a kidney condition, a liver condition, obesity, or a weakened immune system caused by a chronic illness or by medicines taken for a chronic illness. Women who were missing information (223) were excluded from analysis.

Receipt of offer or referral for vaccination from a health care provider was reported by 73.3% of respondents for influenza vaccine and 76.0% of respondents for Tdap (Table 1); among those who received an offer or referral, 65.7% received influenza vaccine, and 70.5% received Tdap. Vaccination offers or referrals were less commonly reported for both influenza vaccine and Tdap by black women and unmarried women (Table 2). Offers or referrals for influenza vaccine were reported less often by women with a college degree or less education, uninsured women, women living in the South, women living below the poverty level, women without other high-risk medical conditions, and women with 10 or fewer health care visits since July 2018. Offers or referrals for Tdap were less frequently reported among women aged 35–49 years, working women, and women with the highest or lowest education levels. Among women reporting offers or referrals for vaccination, vaccination receipt varied by demographic characteristics, with some of the largest gaps in coverage for either vaccine (>20 percentage points) identified between black and non-Hispanic white (white) women. Influenza vaccination coverage was 28 percentage points lower among uninsured women than among privately insured women; sample size was inadequate to analyze this for Tdap.

**TABLE 2:** Influenza vaccination and tetanus toxoid, reduced diphtheria toxoid, and acellular pertussis vaccine (Tdap) coverage among pregnant women who reported a recommendation and offer or referral for vaccination by their health care provider, by selected characteristics — Internet panel survey, United States, March–April 2019

Characteristic	Influenza*			Tdap		
	No.	% (weighted) offered/referred for vaccination	% (weighted) vaccinated among those offered/referred	No.	% (weighted) offered/referred for vaccination	% (weighted) vaccinated among those offered/referred
**Total**	**2,067**	**73.3**	**65.7**	**817**	**76.0**	**70.5**
**Age group (yrs)**						
18–24	441	69.8^†^	65.4	155	80.0^†^	70.1^†^
25–34	1,153	74.4	64.7	480	78.0^†^	72.5^†^
35–49^§^	473	74.9	68.5	182	65.0	63.8
**Race/Ethnicity^¶^**						
White, non-Hispanic^§^	1,252	74.2	69.0	542	78.2	77.4
Black, non-Hispanic	230	69.0^†^	46.6^†^	87	68.3^†^	53.3^†^
Hispanic	365	74.3	70.8	113	75.2	66.1^†^
Other, non-Hispanic	220	75.6	72.3	75	82.1	66.7^†^
**Education**						
Less than high school diploma	511	69.7^†^	58.4^†^	205	73.1	65.7^†^
Some college, no degree	480	70.5^†^	60.2^†^	206	78.8^†^	68.6^†^
College degree (2- or 4-year)	833	75.2^†^	70.3^†^	314	77.6^†^	72.2^†^
More than college degree^§^	243	81.4	75.8	92	70.6	81.7
Marital status**						
Married^§^	1,224	76.7	73.7	547	78.2	73.2
Unmarried	842	69.0^†^	54.4^†^	270	72.6^†^	65.8^†^
**Employment status^††^**						
Working^§^	1,166	75.3	69.5	396	72.7	69.0
Not working	901	70.8	60.4^†^	421	79.1^†^	71.7
**Poverty status^§§^**						
At or above poverty^§^	1,596	75.4	68.5	624	76.9	72.5
Below poverty	469	66.8^†^	55.4^†^	192	74.2	63.9^†^
**Area of residence^¶¶^**						
Nonrural^§^	1,670	73.8	66.4	638	76.6	70.1
Rural	397	71.2	61.9	179	73.9	72.2
Region***						
Northeast^§^	340	78.4	67.9	126	74.8	75.5
Midwest	483	74.7	67.3	206	78.5	72.8
South	844	69.9^†^	63.2	335	75.6	66.7^†^
West	400	73.8	66.2	150	75.4	70.8
**Prenatal insurance status^†††^**						
Private/Military^§^	1,035	76.4	73.1	410	77.4	77.0
Public	955	71.9	58.9^†^	389	75.4	65.2^†^
Uninsured	77	51.0^†^	45.1^†^	<30	—^§§§^	—^§§§^
**No. of provider visits since July 2018**						
None	N/A	N/A	N/A	N/A	N/A	N/A
1–5	395	63.4^†^	60.1^†^	N/A	N/A	N/A
6–10	784	72.8^†^	67.4	N/A	N/A	N/A
>10^§^	888	78.0	66.2	N/A	N/A	N/A
**High-risk condition for influenza^¶¶¶^**						
Yes^§^	886	78.4	65.4	N/A	N/A	N/A
No	971	69.8^†^	66.7	N/A	N/A	N/A

**Abbreviation:** N/A = not applicable.

* Women who did not report any provider visits since July 2018 (30) were excluded from the influenza analysis as they could not have received a provider offer of or referral for vaccination during influenza season. No women were excluded from the Tdap analysis.

^†^ ≥5 percentage-point difference compared with referent group.

^§^ Referent group for comparison within subgroups.

^¶^ Race/ethnicity was self-reported. Women identified as Hispanic might be of any race. The “other” race category included Asians, American Indians or Alaska Natives, Native Hawaiians or other Pacific Islanders, and women who selected “other” or multiple races.

** Excludes one woman who did not report marital status.

^††^ Women who were employed for wages and self-employed were categorized as working; those who were out of work, homemakers, students, retired, or unable to work were categorized as not working.

^§§^ Poverty status was defined based on the reported number of people and children living in the household and annual household income, according to the U.S. Census poverty thresholds (https://www.census.gov/data/tables/time-series/demo/income-poverty/historical-poverty-thresholds.html).

^¶¶^ Rurality was defined using ZIP codes where >50% of the population resides in either a nonmetropolitan county and/or a rural U.S. Census tract, according to the Health Resources and Services Administration’s definition of rural population (https://www.hrsa.gov/rural-health/about-us/definition/index.html).

*** *Northeast*: Connecticut, Maine, Massachusetts, New Hampshire, New Jersey, New York, Pennsylvania, Rhode Island, and Vermont. *Midwest*: Illinois, Indiana, Iowa, Kansas, Michigan, Minnesota, Missouri, Nebraska, North Dakota, Ohio, South Dakota, and Wisconsin. *South*: Alabama, Arkansas, Delaware, District of Columbia, Florida, Georgia, Kentucky, Louisiana, Maryland, Mississippi, North Carolina, Oklahoma, South Carolina, Tennessee, Texas, Virginia, and West Virginia. *West*: Alaska, Arizona, California, Colorado, Hawaii, Idaho, Montana, Nevada, New Mexico, Oregon, Utah, Washington, and Wyoming.

^†††^ Women pregnant on their survey date were asked about current insurance; women who had already delivered were asked about insurance “during your most recent pregnancy.” Women considered to have public insurance selected at least one of the following when asked what kind of medical insurance they had: Medicaid, Medicare, Indian Health Service, state-sponsored medical plan, or other government plan. Women considered to have private/military insurance selected private medical insurance and/or military medical insurance and did not select any type of public insurance.

^§§§^ Estimates with sample size <30 are not reported.

^¶¶¶^ Conditions other than pregnancy associated with increased risk for serious medical complications of influenza include chronic asthma, a lung condition other than asthma, a heart condition, diabetes, a kidney condition, a liver condition, obesity, or a weakened immune system caused by a chronic illness or by medicines taken for a chronic illness. Women who were missing information (223) were excluded from analysis.

The most commonly reported primary reason for not receiving influenza vaccination was believing the vaccine is not effective (17.6%) (Supplementary Figure, https://stacks.cdc.gov/view/cdc/81478). For Tdap, the most commonly reported primary reason for nonvaccination was not knowing vaccination is needed during each pregnancy (37.9%): 24.5% of women who were not vaccinated during their recent pregnancy reported previous receipt of Tdap, and 13.4% reported not knowing they were supposed to receive Tdap during their recent pregnancy. For both vaccines, the second most common reason for nonvaccination was concern about safety risks to their infant (influenza = 15.9%; Tdap = 17.1%).

## Discussion

Eight years of surveillance data corroborate earlier findings (*1*–*3*) regarding the disproportionate burden of influenza-associated hospitalization among pregnant women as well as influenza- and pertussis-associated hospitalization among infants too young to be vaccinated. Approximately half of pregnant women in the United States received influenza vaccine during the 2018–19 influenza season, and findings were similar for Tdap. Approximately three quarters of pregnant women reported an offer or referral for either vaccine from a health care provider, and vaccination coverage was higher among women reporting receipt of an offer or referral. However, ≥30% of women whose providers did offer or refer them for vaccination remained unvaccinated.

Whereas approximately 9% of U.S. females aged 15–44 years are pregnant at any given time each year,^†††^ pregnant women in this age group accounted for 24%–34% of influenza-associated hospitalizations per season. Influenza vaccination reduces pregnant women’s risk for influenza-associated hospitalization by an average of 40% (*7*); maternal vaccination also reduces influenza-associated hospitalization risk in infants aged <6 months by an average of 72% (*5*). Third-trimester maternal Tdap vaccination is 77.7% effective in preventing pertussis cases and 90.5% effective in preventing pertussis hospitalizations in infants aged <2 months (*6*), who account for half of all infant pertussis hospitalizations. Infant protection can motivate pregnant women to receive recommended vaccines, and intention to vaccinate is higher among women who perceive more serious consequences of influenza or pertussis disease for their own or their infant’s health (*11*). It is important to emphasize the well-documented effectiveness of maternal vaccination in preventing the most severe outcomes of influenza and pertussis infection, particularly among very young infants, in patient-facing materials and discussions promoting vaccination during pregnancy. The second most common reason for not receiving either vaccine was concerns about safety risks posed to the fetus, yet studies consistently affirm the safety of maternal vaccination for women and infants (*5*,*8*,*9*). Providers treating pregnant women can take advantage of resources from CDC^§§§^ and provider organizations^¶¶¶^ to help convey strong, specific recommendations for influenza and Tdap vaccination in a manner that is responsive to women’s concerns.

Consistent with prior findings (*10*,*12*), current survey data show that vaccination coverage was lower among black pregnant women and those of lower socioeconomic status (i.e., less educated, living in poverty, and publicly insured or uninsured). Because provider recommendations are a powerful predictor of vaccination among pregnant women (*10*,*11*), previous efforts have focused on encouraging providers to strongly recommend needed vaccines and either offer them or provide referrals to another vaccinator if vaccines are not stocked onsite (*13*). This analysis found overall high levels of reported provider offers or referrals for both vaccines although differences in some demographic subgroups were noted. Lower reported provider offers or referrals were sometimes associated with lower vaccination coverage. Further, many women whose providers offered or referred them for vaccination remained unvaccinated. This finding was particularly striking among black women, fewer than half of whom (46.6%) accepted influenza vaccine when offered or referred, compared with approximately two thirds (69.0%) of white women; similarly, Tdap coverage was 53.3% among black women, compared with 77.4% among white women (and 66.1% among Hispanic women), offered or referred for vaccination. One study in the general population found that black adults had lower levels of trust in influenza vaccine, in their doctor, and in information from CDC, compared with white adults (*14*); similar beliefs among black pregnant women might explain the lower vaccine acceptance found in this analysis. Differential effects of provider vaccination offers or referrals might also be explained by less patient-centered provider communication with black patients (*15*).

Surveillance and survey data presented here are subject to several previously described limitations that might affect their representativeness (*10*,*16*–*18*). Importantly, surveillance data likely underestimate outcomes of interest, while self-reported vaccination data might under- or overestimate true coverage. In addition, respondents excluded from Tdap coverage analysis differed from those included on race/ethnicity, education level, insurance type, poverty status, and region of residence.

These findings highlight influenza and pertussis disease burden among pregnant women and infants and vaccination coverage among pregnant women in the United States and suggest that disease burden could be reduced by improving vaccination coverage. Many pregnant women do not receive both vaccines recommended during pregnancy, increasing their and their newborns’ risk for influenza and pertussis infection and their potentially devastating consequences. Although pregnant women differ in responses to vaccination offers and referrals, health care providers remain their most trusted source of vaccine information (*11*). Starting maternal vaccination discussions with patients early in pregnancy can offer providers multiple opportunities to share information tailored to individual patients’ needs and address vaccination-related concerns.

**Summary**

**What is already known about this topic?**

Vaccinating pregnant women with influenza vaccine and tetanus toxoid, reduced diphtheria toxoid, and acellular pertussis vaccine (Tdap) can reduce their own risk for disease and protect their young infants against influenza and pertussis.

**What is added by this report?**

Influenza and pertussis cause substantial disease burden among pregnant women and infants too young to be vaccinated. Approximately half of pregnant women reported receiving each vaccine. Even among pregnant women reporting vaccination offers or referrals from a health care provider, approximately one third remained unvaccinated.

**What are the implications for public health practice?**

CDC and provider organizations’ resources are available to help providers convey strong, specific recommendations for influenza and Tdap vaccination that are responsive to pregnant women’s concerns.

## Footnotes

* In this report, influenza burden is defined as the total number and seasonal range of influenza-associated hospitalizations among pregnant women and influenza-associated deaths in infants aged <6 months, the proportion of influenza-associated hospitalizations among women of childbearing age occurring among pregnant women, and the influenza-associated hospitalization rate per 100,000 population for infants aged <6 months. Pertussis burden is defined as the number of cases, reported hospitalizations, and deaths among children aged <1 year and the proportion of each of these occurring among infants aged <2 months.

† Descriptions of CDC’s influenza surveillance systems are available at https://www.cdc.gov/flu/weekly/overview.htm.

§ https://wwwn.cdc.gov/nndss/. Detailed annual reports of pertussis surveillance data are available at https://www.cdc.gov/pertussis/surv-reporting.html.

¶ https://www.dynata.com.

** An opt-in Internet panel survey is a nonprobability sampling survey. The denominator for a response rate calculation cannot be determined because no sampling frame with a selection probability is involved at the recruitment stage. Instead, the survey cooperation rate is provided.

†† Among 2,626 respondents, 2,098 (79.9%) were pregnant any time during October 2018–January 2019. One respondent did not provide her influenza vaccination status and was excluded from analysis. Therefore, influenza vaccination coverage before or during pregnancy was assessed among 2,097 respondents.

§§ Among 2,626 respondents, 1,494 (56.9%) were still pregnant at the time of the survey, and 202 (7.7%) reported a pregnancy outcome other than live birth. Among 930 respondents reporting a live birth, 113 women (12.8%) who reported not knowing if they had received Tdap ever (10.3%) or during their recent pregnancy (2.5%) were excluded from analysis. Therefore, Tdap receipt during pregnancy was assessed among 817 respondents.

¶¶ Additional information on obstacles to inference in nonprobability samples is available at https://www.aapor.org/AAPOR_Main/media/MainSiteFiles/NPS_TF_Report_Final_7_revised_FNL_6_22_13.pdf and https://www.aapor.org/getattachment/Education-Resources/For-Researchers/AAPOR_Guidance_Nonprob_Precision_042216.pdf. Although the estimates reported here have variance, there has been no attempt to quantify the size of the variance.

*** The proportion of FluSurv-NET cases with known pregnancy status was not ascertained during the 2010–11 influenza season. Pregnancy status was known for 88% of females aged 15–44 years reported to FluSurv-NET during the 2011–12 influenza season and >99% during the 2012–13 through 2017–18 seasons.

††† Based on 2014 estimates of the pregnancy rate from https://www.guttmacher.org/report/pregnancy-desires-and-pregnancies-state-level-estimates-2014 and U.S. Census population estimates of U.S. females aged 15–44 years in 2014 (https://data.census.gov/cedsci/table?q=females%2015-44&hidePreview=false&table=S0101&tid=ACSST1Y2014.S0101&lastDisplayedRow=20); approximately 9% of reproductive-aged women in the United States are pregnant at any time.

§§§ https://www.cdc.gov/pertussis/materials/hcp.html.

¶¶¶ http://immunizationforwomen.org/providers/pregnancy/pregnancy-resources.php.
